# A Combined Multidisciplinary Intervention for Health Promotion in the Workplace: A Pilot Study

**DOI:** 10.3390/jcm10071512

**Published:** 2021-04-05

**Authors:** Venerando Rapisarda, Emanuele Cannizzaro, Martina Barchitta, Ermanno Vitale, Diana Cinà, Fabrizia Minciullo, Serena Matera, Massimo Bracci, Antonella Agodi, Caterina Ledda

**Affiliations:** 1 Occupational Medicine, Department of Clinical and Experimental Medicine, University of Catania, 95123 Catania, Italy; vrapisarda@unict.it (V.R.); serena.matera@yahoo.it (S.M.); cledda@unict.it (C.L.); 2 Department of Sciences for Health Promotion and Mother and Child Care “Giuseppe D’Alessandro”, University of Palermo, 90127 Palermo, Italy; emanuele.cannizzaro@unipa.it; 3 Department of Medical and Surgical Sciences and Advanced Technologies “GF Ingrassia”, University of Catania, Via Santa Sofia 100, 95123 Catania, Italy; martina.barchitta@unict.it (M.B.); agodia@unict.it (A.A.); 4 Health Management of the “Cannizzaro” Emergency Hospital of Catania, 95126 Catania, Italy; dianacinact@gmail.com; 5 Department of Prevention, Food Hygiene Service, Nutritional Surveillance and Prevention, Provincial Health Authority of Catania, 95027 San Gregorio di Catania, Italy; fabrizia.minciullo@hotmail.it; 6 Occupational Medicine, Department of Clinical and Molecular Sciences, Polytechnic University of Marche, 60126 Ancona, Italy; m.bracci@univpm.it

**Keywords:** workplace health promotion, healthcare workers, cardiovascular risk factor, work ability index (WAI), Mediterranean diet, body image dissatisfaction (BID)

## Abstract

The aim of this study was to assess the effects of a joint health promotion intervention on a cohort of healthcare workers (HCWs) who had at least one cardiovascular risk factor. The HCWs were assessed at three different times, i.e., time zero (T0), after 6 months (T6), and after 12 months (T12). The following parameters were measured at a medical examination: physical activity, blood pressure, waist circumference, body mass index (BMI), routine laboratory tests, plicometric analysis, work ability index (WAI), and body image dissatisfaction (BID). Among the 447 HCWs, 38 HCWs were included in the study; 45% (n = 17) were male. At T12, the average blood pressure, waist/hip ratio (WHR) index, BMI, total cholesterol, triglyceride level, and blood glucose values were reduced. The levels of physical activity and adherence to the Mediterranean diet had progressively increased. The WAI showed a significant shift from low to good work performance at T12, as well as BID score. This is the first study that has analyzed work performance in relation to a workplace health promotion through a multidisciplinary approach. This health promotion intervention that combined diet and sport activity has led to a significant change in HCWs’ lifestyles and body perceptions, as well as their ability to work. This project highlights the importance of using a multidisciplinary approach and the workplace setting in health promotion programs.

## 1. Introduction

Diet plays a key role in human life for maintaining health and strength; however, food can also be the cause of several diseases [1,2,3].

The daily energy needs of a worker depend on many factors such as anthropometrics, job, environment, and hours of work [4,5,6,7,8,9,10,11].

Studies have shown that poor or rich diets can cause workers to lose 20% of their productivity [12]. Following modest investments to improve nutrition at work, the impact in terms of reduction in sick days and accidents at work can be significant [9,10,11,12]. In fact, a poor diet (high calorie, unbalanced, or low-calorie diets) has been included among the risk factors for many pathologies, in particular, metabolic syndrome, obesity, cardiovascular disease, diabetes, deficiency syndrome, gastrointestinal disease, and neoplasms [13,14]. Obese subjects present a higher risk of developing occupational accidents and diseases as some occupational risks may also be amplified in relation to reduced mobility/resistance of these subjects [15]. First, the maintenance of prolonged fixed postures can lead to early onset of musculoskeletal system diseases such as spondylosis, discopathies, and osteoarthritis of the knee. Furthermore, obesity is an additional risk factor for injury, due to important physical limitations in movement and agility, which can reduce worker safety [11,12]. Metabolic syndrome is a clinical situation in which several interrelated factors contribute to increasing the possibility of developing diseases affecting the cardiovascular system and metabolism (e.g., diabetes) [16,17,18,19,20,21].

Work activities, which often present chemical, physical, and biological, as well as accident and organizational risk factors, can negatively impact the development of a disease and related complications [11,12]. 

In fact, workers with pathologies such as diabetes, obesity, and neoplastic diseases are more exposed to risk factors present in work milieus [2,4,9,10]. Health care workers (HCWs) are among those with the highest health and safety risks. Indeed, HCWs are constantly exposed to biological hazards, load, and patient transfers, as well as work shifts and the stress of caring for their patients all the time [22,23,24,25,26].

Unhealthy nutrition at work may be the result of easy access to unhealthy food (vending machines, fast foods, and take-out); difficulty finding places to eat at work; difficulty preparing/heating food (microwaves, electric ovens, etc.); difficulty storing food (fridge, cupboards, etc.); and difficulty retrieving nutritional information in canteens and vending machines [27,28]. There is strong evidence that interventions in the workplace are effective both for fostering the consumption of fruit, vegetables, and fiber-rich foods and for decreasing the amount of fat intake among adults [6].

The aim of this study was to assess the short- and medium-term (six- and twelve-month study) effects of a joint health promotion intervention aimed at improving lifestyles, i.e., diet and physical activity and the metabolic and anthropometric measures of a cohort of HCWs who had at least one cardiovascular risk factor.

## 2. Materials and Methods

### 2.1. Population

Participation in this study was offered to the HCWs at an Emergency Hospital, who had undergone mandatory health surveillance, pursuant to Law Decree (DL) 81/08, from January to May 2019. All HCWs invited to take part in the project were informed about the objectives and procedures of the study. It was not necessary to receive confirmation from the ethical committee as the activity is ruled by the Law Decree (DL) 81/08 article 25 within the health promotion actions. Participation in the study was on a voluntary basis and each participant gave informed written consent. The workers’ council was involved in the study design. The inclusion criteria were as follows:

- overweight or obesity, i.e., body mass index (BMI) >25, or waist circumference >102 cm (males), >88 cm (females);
- dyslipidemia without pharmacological treatment, i.e., total cholesterol >220 mg/dL, or HDL cholesterol (high-density lipoprotein) <35 mg/dL, or low-density lipoprotein cholesterol (LDL) >130 mg/dL, or triglycerides >200 mg/dL;
- fasting glucose levels >120 mg/dl and/or reduced tolerance to glucose or diabetes mellitus, without pharmacological treatment was determined through HbA1c.
- 10 mL of peripheral blood were drawn in the morning after a fasting night to determine haematological parameters.

The exclusion criteria were refusal of written informed consent; diabetes mellitus under pharmacological treatment; dyslipidemia under pharmacological treatment; recent cancer diagnosis; pregnancy; or chronic diseases such as kidney failure, heart disease, uncompensated endocrine disorders. No age limits were applied.

### 2.2. Clinical Parameters

Each person underwent a medical examination, including history of eating habits, objective examination, weight, and height measurement for the calculation of BMI, and other anthropometric parameters for a waist/hip ratio (WHR) index assessment.

Subjects were tested at time zero (T0), after 6 months (T6) and after 12 months (T12). The following parameters were measured during the medical examination: (i) Physical activity was measured in terms of type, frequency (days/week), and duration (in minutes) through a self-questionnaire. To ascertain the energy consumption produced by physical activity, the data were converted into their metabolic equivalent (MET) [29]. The total weekly caloric consumption was calculated using the formula of the International Physical Activity Questionnaire (IPAQ) [30]. Each subject was also required to say whether the activity performed really coincided with the experimental period (Σ (MET × frequency × duration)). (ii) For blood pressure, systolic and diastolic blood pressures (mmHg) were measured three times using the left arm, with the subject seated and at rest for 5 min. The averages of the second and third readings were recorded. (iii) Waist circumference was measured to the nearest centimeter, using a flexible meter, at the end of exhalation, positioning the meter at navel level [31]. (iv) For the BMI, height and weight were measured, with the subject barefoot and lightly dressed and their body mass index was calculated according to the formula weight (kg) divided by height (m) squared. (v) The routine laboratory tests that were performed included liver and kidney function, cholesterol levels (total, LDL, and HDL), triglycerides, blood sugar, and protidogram. (vi) The plicometric (GIMA Fit. Comp Pli Pli Fitness & Computer, Gessate Milano) and impedance analysis (body fat analyzer, Model BT905, Skylark Tokyo, Japan) were performed to evaluate percentage of fat and lean mass and calculate basal metabolism.

The IPAQ (2004) was used to assess the physical activity of each participant. Each participant was asked to indicate the type, frequency (days per week), and duration (hours or minutes per day) of each physical activity performed during the last seven days. The assessment was based on the intensity of the physical activities classified as vigorous (e.g., aerobic walking, jogging, and running), moderate (e.g., brisk walking, general exercises at home, and recreational swimming), or normal walking. The level of physical activity was classified as low, moderate, or high, based on metabolic energy (MET). Further data were collected using a standardized questionnaire aimed at assessing the degree of adherence to the Mediterranean diet, as validated by Martínez-González et al. [32]. The responses to the questions regarded daily and weekly intake of nutrients such as fruits, vegetables, seasonings (oil), meat, and fat generated a total score. The higher the score, the closer the respondent’s eating habits were to the Mediterranean model, i.e., poor adherence ≤5, average adherence 6–9, and good adherence ≥10.

The work ability index (WAI) was used to assess work ability at T0 and T12. The WAI scores were calculated according to the standard method provided by the (Finnish Institute of Occupational Health (FIOH) [33,34]. The WAI is composed of the following factors: (1) current working ability as compared with the best period of life (0–10 points), (2) ability to work in relation to the demands of a task (2–10 points), (3) number of current diagnoses made by the doctor (1–7 points), (4) reduction in work ability due to disease(s) (1–6 points), (5) absences due to illness in the last 12 months (1–6 points), (6) personal forecast of work ability for the following two years (1.4 and 7 points), and (7) psychological conditions/resources (1–4 points). A total score ranged from 7 to 49. The objective was to detect any changes in work ability in relation to age, gender, pathologies, and the intervention studied (pre- and post-treatment WAI) [34]. The WAI was calculated by adding up the individual points. The higher the score, the better the ability to work. There are 4 different levels, i.e., low (score 7–27), moderate (score 28–36), good (score 37–43), and excellent (score 44–49). HCWs with a WAI score lower than 36 were classified as having low working ability and HCWs with a WAI score higher than 37 were classified as having satisfactory working ability. Therefore, the WAI, with a view to prevention and health promotion, is a screening tool that can give an indication of a worker’s the state of well-being within a work organization [34].

The analyses of self and body dimensions were evaluated using a figure rating scale (FRS) [35] and involved comparing the shape of each HCW’s body with 9 shapes ranging from very thin (FRS = 1) to severely obese (FRS = 9).

Participants were asked to assess how they perceived their current fitness or “how they looked” by choosing a score corresponding to their figure on a scale from 1 to 9. Participants were also asked to indicate the “ideal” figure or “how they would like to look”. The discrepancy between the two figures was recorded as an indication of dissatisfaction with their body image. The body image dissatisfaction (BID) variable was used, calculated by subtracting the current FRS score from the ideal body size FRS score. A BID score ≥1 indicated that the HCW “wished to be thinner”; a BID score <1 indicated that the HCW “wished to be fatter”; a BID score of zero indicated that the HCW were satisfied with their body.

### 2.3. Investigation Timetable

**Figure 1:**
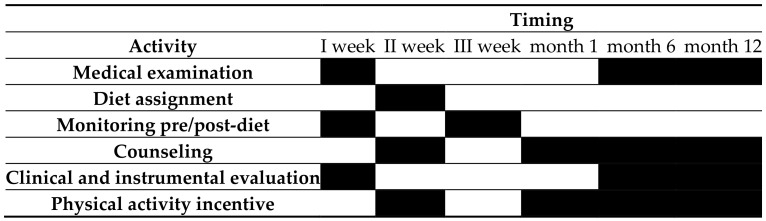
Gantt chart of procedures from time zero (T0) to after 12 months (T12).

The sample of HCWs was recruited from January to May 2019, during the periodical health surveillance, pursuant to Legislative Decree 81/08. Figure 1 shows the Gantt chart of procedures from T0 to T12. Encouragement to perform physical activity was achieved through an easy agreement with the university sport facility; sport education; the suggestion to use smartphone sport-guiding apps, which were greatly appreciated in the early phases of the lockdown; and availability of physicians for telephone consultations.

A week after the medical checkup, with a specific software (Nutrigeo8, Progeo, Ascoli Piceno, Italy) an individualized qualitative/quantitative diet was designed for each subject which established the Kcal/days. The software performed calculations related to food chemistry (qualitative and quantitative characterization), basal metabolism, energy requirements, and food portions. The software draws on an official database containing over 2200 foods and recipes. For 14 days (7 days before diet delivery and 7 days from the start of the diet) all subjects were asked to send via mobile phone/email a photographic diary of all the foods consumed during the day; the first 7 days before the start of the diet were to study the eating habits and the 7 days after the start of the diet were to assess adherence to the prescribed diet.

Four weeks after recruitment, the HCWs were called back for a further individual interview with a nutritionist, who offered personalized advice on dietary habits and physical activity level, during which they discussed the results of the dietary and physical activity diary and their adherence to the Mediterranean diet. At the return interview, the nutritionist, who was also trained in motivational techniques, encouraged participants to identify and set goals for their physical activity and proper nutrition. Using a motivational counselling approach, the HCWs were helped to identify strategies for achieving these goals, by providing them with useful material, such as the food pyramid [36] and brochures on physical exercise and reducing the use of salt in food, published by the Italian Ministry of Health.

The check-in meetings with the nutritionist were scheduled once a month; at each meeting, each participant received additional motivational support that reminded them of their goals and how to change their life habits. After 6 months (T6) from T0, the HCWs met again with the occupational physician involved in the project, who measured anthropometric parameters (weight, height, and waist), requested blood tests, and collected a diary on dietary and physical activity. For the 12-month follow-up (T12), the same procedure as the T6 follow-up was followed.

### 2.4. Statistical Analysis

The data were analyzed using SPSS 22.0 software (SPSS-PC IBM Corp., Armonk, NY, USA). Once the normality of the distribution had been assessed, the quantitative variables were processed using the average and the relative standard deviation from the average, while the qualitative variables were assessed in terms of frequency. For the comparison between the two averages, the Student’s *t*-test was used for paired data. For comparison among several averages the one-way variance analysis (ANOVA) was used. The frequency comparison was performed with Fisher’s exact test. Two-tailed tests were used, with a nominal significance level of *p* < 0.05.

## 3. Results

### Characteristics of the Sample at Time Zero (T0)

**Figure 2:**
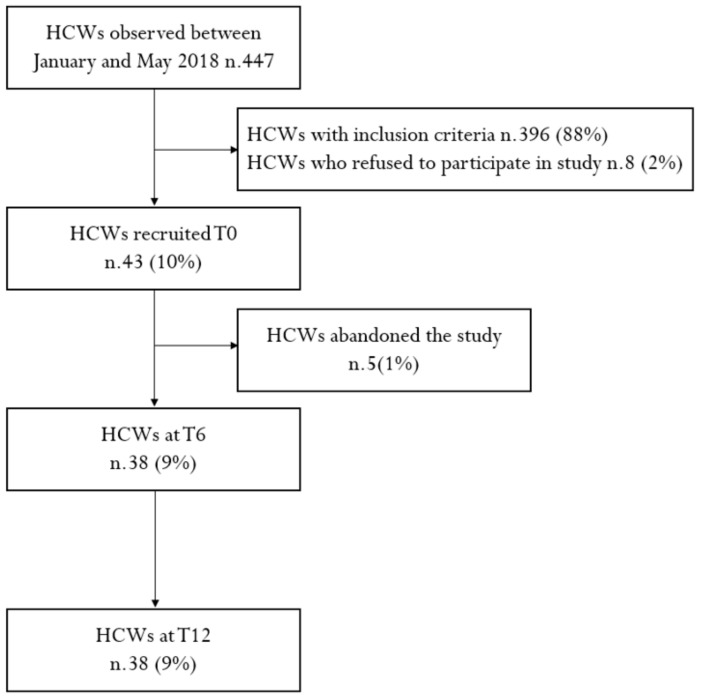
Flowchart of the sample studied illustrating in/excluded studies.

Among the 447 HCWs, who in the five months (January–May) of 2019 underwent health surveillance pursuant to Law Decree 81/08, 396 of the HCWs (88%) were ruled out of the study because they met the exclusion criteria; eight of the HCWs (2%) refused to enter the study; another five HCWs (1%) left the study in the period between T0 and T6, due to lack of time. The eight HCWs who did not agree to participate in the study, gave the following reasons: four HCWs (50%) were already on a low-calorie diet, three HCWs (37%) had no time, and one HCW (13%) wanted no lifestyle limitations. Figure 2 shows a flowchart of the sample studied. Therefore, 38 HCWs were included in the study who met the inclusion criteria based on the following parameters: 12 HCWs (32%) had a BMI >25, 23 HCWs (60%) had a BMI >25 and total cholesterol >220, and 3 HCEs (8%) had a BMI >25, total cholesterol >220, and increased fasting glucose.

The study sample of 38 HCWs (100%) included 45% (n = 17) male HCWs. Male and female groups were homogeneous, all of them worked shifts on all three shifts (see Table 1 and Table 2).

The mean values of diastolic (DBP) and systolic (SBP) blood pressures were within normal range (60 > DBP < 90 mmHg and 100 < SBP < 140 mmHg), for both female and male HCWs.

The average BMI values were within the overweight range (BMI 25–29.9), for both sexes.

The average values of the WHR index were above the normal limits (0.95 for males and 0.80 for females).

The average total cholesterol levels for males were above the normal range (120–220 mg/dL) as compared with females.

The average HDL (40–80 mg/dL) and LDL (70–180 mg/dL) cholesterol levels were within normal ranges, with no difference between the two sexes.

Triglyceride and blood sugar levels were within normal range, although the average values were high. Finally, total calorie consumption was higher for male than female HCWs, but not significantly higher. It should be noted that only 24% (n = 4) of the male HCWs and 29% (n = 6) of the female HCWs were engaged in sports.

Follow-up was performed after six (T6) and twelve months (T12). Table 2 shows the results observed at T0, T6 (after 6 months) and T12 (after 12 months), broken down by gender.

The average SBP and DBP values decreased during the time period, i.e., SBP significantly in males from T0 to T12 and DBP significantly in females from T0 to T12.

Weight was recorded, detectable as a substantial, but not significant, reduction in waist size, from T0 to T12 for both male and female HCWs, as was the WHR index.

At the same time, the average BMI of both male and female HCWs was significantly reduced from T0 to T12.

Regarding metabolic variables, the intervention led to a significant reduction in total cholesterol for both male and female HCWs from T0 to T12. The HDL levels increased, while the LDL levels decreased, but were not statistically significant.

The triglyceride levels gradually decreased, but not significantly.

The average blood glucose values decreased from T0 to T12 and were statistically significant for females as compared with male HCWs.

Finally, as far as sport activity was concerned, everyone embarked on a sporting activity, changing their lifestyle. The levels of physical activity, measured in energy consumption, progressively increased and was statistically significantly for female as compared with male HCWs.

Adherence to the Mediterranean diet, evaluated at T0 and T12, significantly (*p* = 0.001) increased for both in male and female HCWs (4.6 ± 2.3 vs. 7.7 ± 1.7 and 4.8 ± 1.9 vs. 7.9 ± 1.9, for males and females, respectively) (Table 3).

The average results of the WAI showed a significant shift from low work performance at T0 to good work performance at T12, for both male and female HCWs.

The analysis of self and body dimensions enabled us to observe at T0 the “desire to be thinner”, in the entirety (100%) of the sample. Other motivations which urged workers to participate were reduce cholesterol levels (15 HCWs or 39%) and to be more athletic (8 HCWs or 21%).

At T12, 71% (n = 12) of male HCWs and 62% (n = 13) of female HCWs were “satisfied with their bodies”, while the rest remained in need of losing more weight although no adverse physical results were observed.

## 4. Discussion

The cohort examined was recruited as part of the mandatory health surveillance, pursuant to LD 81/08 which all Italian workers must comply with. In fact, all Italian health care workers who are exposed to biological as well as shift work health risks, must undergoing annual, mandatory check-ups in order to obtain work suitability. The HCWs who met the eligibility characteristics were 10% (n = 43) of the entire population studied (n = 447). These data is in line with that of the Region of Sicily on obesity (www.epicentro.iss.it (accessed on 30 January 2021)).

In this study, only 3% of the sample did not continue the follow-up. This was extremely encouraging and testifies to the effectiveness of the proposed multidisciplinary approach as compared with other studies where dropout rates have been over 20% [37,38]. The drop-out rates observed were similar to those of Christensen et al. [39]. A possible explanation for our results may be the small size of the hospital where the study was implemented; the hospital, in this study, has 1500 workers, including administrative staff, who operate within a single Hospital Praesidium, in well-connected pavilions with high standards in internal communications among the various operating units. This probably helped with communications to the HCWs and with the possibility of setting appointments and work commitments.

The health promotion intervention provided to the HCWs was characterized by working on diets and physical activity evaluation with the outcomes including work performance and perception of one’s own image.

As noted in previous studies, the combined interventions seem to lead to better results than individual interventions [37,40,41,42].

The results of this study are promising, especially the effects recorded on participants’ weight reduction and improvement of their lipid profile, blood pressure, work performance, and self-image perception. There was a considerable increase in the level of physical activity and Kilocalories burned during exercise at T12 as compared with at T0. Among all the HCWs, dietary habits together with the individualized dietary program designed for each worker were associated with a significant reduction in BMI at T12 and, in statistically insignificant ways, with reductions in waist size and the WHR index at T12 as compared with at T0. This result is in line with other studies where an improvement in metabolic variables with respect to waist circumference was highlighted [37,43].

Blood monitoring of the lipid profile showed a significant reduction in total cholesterol, LDL cholesterol, and triglycerides at T12 as compared with at T0.

The HDL cholesterol levels remained virtually unchanged. In other studies, reductions in HDL levels were observed along with total and LDL cholesterol [37].

Average blood pressure at T12 also improved progressively as compared with at T0, moving from defined prehypertension values to significantly lower values for SBP in males and DBP in females. Similar results were found by Scapellato et al. [37] who followed a cohort of HCWs, also with a combined action including diet and physical activity.

This study, with the intent of being a pilot study, is innovative in that it introduces the evaluation of work performance. The results show that work performance scores (WAI) at T12 significantly improved as compared with T0.

Metabolic variables such as lipid profile (total and LDL cholesterol and triglycerides), SBP, DBP, BMI, WHR index, and waist circumference showed an overall improvement in both sexes.

These results have been wholly or partly achieved in similar studies [37,39]. The WHR index is a predictive cardiovascular risk factor. In our study, the WHR index was reduced, although not statistically significantly, for all HCWs. Other studies have reported statistically significant changes in the WHR index following intervention [39]. This difference is explained by the fact that the initial circumference of the sample studied by Christensen et al. [39] was much larger than our sample.

A study conducted by Mecca et al. [44] showed that a reduction in waist circumference seemed to be due almost exclusively to the diet adopted and, in particular, to the increased intake of fruit, vegetables, and fiber.

Exercise significantly improved both in terms of Kcals consumed and in the number of males and females participating in sports.

Some studies have hypothesized a role of physical activity performed during work activity in calorie consumption and weight loss [37]. However, most studies have shown that the biological effects of different types of physical exercise on weight and cardiovascular risk, sport being compared to work activity, differed [45,46]. Indeed, it has been shown that an increased amount of physical activity at work is not associated with a reduction in BMI or cardiovascular risk. Work involving higher levels of physical exertion may also have a negative effect, due to the fact that people are less inclined to exercise in their free time [47,48,49].

Increased physical demand in the workplace can also increase the sense of hunger, inducing a higher caloric intake, resulting in weight gain rather than weight loss [46].

Regarding metabolic factors, the health promotion intervention led to reductions in total and LDL levels, triglyceride levels, as well as SBP and DBP. These results are in line with the findings of Pattyn et al. [50] and Blackford et al. [51].

The reduction in triglycerides, as reported by Ahmed et al. [52] and Blackford et al. [46], also seems to be linked to a moderate increase in physical activity. In our study, we found a concomitant improvement in physical activity and triglyceride levels in both males and females.

A meta-analysis by [53] confirmed the influence of (aerobic) exercise on triglyceride levels, particularly on overweight/obese adults.

Blood glucose levels at T12 improved considerably, for both males and females, but in a statistically non-significant way. As in our study, Groeneveld et al. [33] also reported an improvement in blood glucose levels after similar interventions.

Finally, this study also assessed the effect that weight reduction had on each participant’s perception of their body. The results showed that over 60% of all HCWs were satisfied with their bodies. Similar results were found in a study conducted by Radwan et al. [54], who found a close correlation between BMI and BID scores.

In Italy, a research group coordinated by Agodi showed poor adherence to the Mediterranean Diet especially in women of childbearing age [55,56,57,58,59].

The strengths of this study were the combined approach with sports and diet and multidisciplinary activities, through the involvement of trained technical staff, exploiting the skills of several disciplines (dietetics, occupational medicine, public hygiene, sport medicine, etc.) for achieving a common goal. Additional strengths included the high adherence, by all HCWs to the Mediterranean diet and sport activities and the very low dropout rate (about 3%); notably, less than 10% of the total study population was examined.

The main weaknesses of the study included the small sample size, the limited follow-up of only 12 months, and the lack of a control group.

## 5. Conclusions

The objective of improving health and well-being in the workplace may be achieved through the joint efforts of employers, workers, and prevention professionals, including occupational physicians. This pilot study reports the preliminary results of a WAI campaign which, through the improvement of workers’ eating habits, has led to better metabolic parameters, BMI, work ability, and body perception by each participating subject. Therefore, this study confirms that the working environment is a place where it is possible to develop “health promotion” programs. [60,61,62]. The lifestyles, understood as sports and eating habits, of the HCWs participating in this study improved statistically from T0 to T12. Combined diet and sport activity led to significant lifestyle changes and improved body perception and ability to work.

The positive outcomes of this study should encourage occupational doctors to also promote HCWs’ health, while also preventing specific risks that HCWs are exposed to all the time.

This project highlighted the importance of using a multidisciplinary approach in health promotion programs and the role that occupational medicine and hospital hygiene services can play for improving HCWs’ health. This program could also encourage other companies to propose similar interventions for improving the lifestyles of their employees. Indeed, in order to assess the effects of the proposed intervention, the study should be proposed to a wider range of stakeholders and the follow-up period should be extended to include medium-term effects.

## Figures and Tables

**Table 1:** Features of the sample at T0, divided by gender.

	Males (n = 17)	Females (n = 21)	Value of *p*
**Age (years)**	48.5 ± 7.4	49.3 ± 7.9	n.s.
** Cigarette Packages/year **	5.1 ± 10.6	4.8 ± 9.2	n.s.
**Medical doctor**	3 (17%)	4 (19%)	n.s.
**Technician**	4 (24%)	3 (14%)	n.s.
**Nurses**	10 (59%)	14 (67%)	n.s.
**Working seniority**	13.4 ± 4.6	14.2 ± 4.9	n.s.
**Area of involvement**	clinical (n = 7); surgical (n = 7), Services (n = 3)	clinical (n = 6); surgical (n = 10), Services (n = 5)	n.s.

Significatively was assessed through Student’s *t*-test and Fisher test.

**Table 2:** Data obtained at T0, after 6 months (T6), and T12, broken down by gender.

	T0			T6				T12					
	Males	Females	Gender Differences	Males	Females	Males T0 vs. T6	Females T0 vs. T6	Males	Females	Males T6 vs. T12	Females T6 vs. T12	Males T0Vs T12	Females T0 vs. T12
**SBP (mmHg)**	138.1 ± 13.8	132.4 ± 11.7	n.s.	131.3 ± 11.6	128.5 ± 12.3	n.s.	n.s.	128.3 ± 12.4	126.1 ± 10.1	n.s.	n.s.	<0.05 *	n.s.
**DBP (mmHg)**	88.5 ± 6.9	86.2 ± 6.4	n.s.	86.4 ± 6.3	83.1 ± 5.3	n.s.	n.s.	85.7 ± 6.1	82.5 ± 4.8	n.s.	n.s.	n.s.	<0.05 *
**Waist circumference (cm)**	97.6 ± 7.5	88.5 ± 9.3	<0.05 *	96.4 ± 6.1	86.9 ± 7.8	n.s.	n.s.	95.1 ± 4.7	86.1 ± 6.7	n.s.	n.s.	n.s.	n.s.
**BMI (cm2/kg)**	26.9 ± 3.9	26.8 ± 5.1	n.s.	25.8 ± 5.2	25.4 ± 4.9	n.s.	n.s.	24.2 ± 3.7	24.1 ± 2.8	n.s.	n.s.	<0.05 *	<0.05 *
**WHR index**	0.97 ± 0.09	0.91 ± 0.06	n.s.	0.92 ± 0.87	0.89 ± 0.97	n.s.	n.s.	0.90 ± 0.77	0.88 ± 0.81	n.s.	n.s.	n.s.	n.s.
**Total cholesterol (mg/dL)**	221.8 ± 29.6	218.7 ± 24.9	n.s.	220.4 ± 27.3	211.3 ± 25.4	n.s.	n.s.	203.2 ± 20.3	204.1 ± 19.6	<0.05 *	n.s.	<0.05 *	<0.05 *
**HDL cholesterol (mg/dL)**	56.3 ± 13.2	60.1 ± 15.9	n.s.	56.4 ± 13.6	61.1 ± 14.8	n.s.	n.s.	56.9 ± 11.4	61.9 ± 14.4	n.s.	n.s.	n.s.	n.s.
**LDL cholesterol (mg/dL)**	147.7 ± 21.4	146.6 ± 23.5	n.s.	143.5 ± 20.2	142.2 ± 21.4	n.s.	n.s.	140.2 ± 20.3	140.7 ± 20.1	n.s.	n.s.	n.s.	n.s.
**Triglycerides (mg/dL)**	161.4 ± 75.4	155.4 ± 66.5	n.s.	152.8 ± 76.2	152.1 ± 63.1	n.s.	n.s.	148.9 ± 77.1	148.3 ± 57.2	n.s.	n.s.	n.s.	n.s.
**Blood glucose (mg/dL)**	98.2 ± 11.3	96.4 ± 10.9	n.s.	96.5 ± 10.4	92.1 ± 9.8	n.s.	n.s.	93.1 ± 9.4	89.3 ± 9.4	n.s.	n.s.	n.s.	<0.05 *
**Physical activity (n. sub.)**	4 (24%)	6 (29%)	n.s.	15 (88%)	21 (100%)	<0.05 *	<0.05 *	17 (100%)	21 (100%)	n.s.	n.s.	<0.05 *	<0.05 *
**Physical activity (MET)**	486.9 ±317.5	299.9 ±101.3	<0.05 *	541.1 ±219.4	358.8 ±180.4	n.s.	n.s.	615.6 ±345.1	417.5 ±234.4	n.s.	n.s.	n.s.	<0.05 *

SBP, systolic blood pressure; DBP, diastolic blood pressure; WHR, waist/hip ratio; * Statistically significant difference between two groups.

**Table 3:** Results of adherence to the Mediterranean diet, work ability index (WAI), and body image dissatisfaction (BID), at T0, T6, and T12.

	T0 Males	T12 Males	* p * -Value	T0 Females	T12 Females	*p* -Value
**Adherence to diet**	4.6 ± 2.3	7.7 ± 1.7	0.001 *	4.8 ± 1.9	7.9 ± 1.9	0.001 *
** WAI index **	28.3 ± 7.5	38.2 ± 7.9	0.001 *	27.1 ± 7.5	37.7 ± 6.3	0.001 *
**BID ≥ 1**	17 (100%)	5 (%)	n.s.	21 (100%)	8 (%)	n.s.
**BID < 1**	0	0	n.s.	0	0	n.s.
**BID = 0**	0	12 (71%)	n.s.	0	13 (62%)	n.s.

* Statistically significant difference.

## Data Availability

The datasets used and/or analyzed during the current study are available from the corresponding author on reasonable request.
